# Ablative radiation therapy to restrain everything safely treatable (ARREST): study protocol for a phase I trial treating polymetastatic cancer with stereotactic radiotherapy

**DOI:** 10.1186/s12885-021-08020-2

**Published:** 2021-04-14

**Authors:** Glenn S. Bauman, Mark T. Corkum, Hatim Fakir, Timothy K. Nguyen, David A. Palma

**Affiliations:** 1 Division of Radiation Oncology, Department of Oncology, London Health Sciences Centre, 790 Commissioners Rd. E, London, Ontario N6C 1K1 Canada; 2 Department of Medical Biophysics, London Health Sciences Centre, London, Ontario Canada

**Keywords:** Stereotactic radiotherapy, Cancer, Metastatic disease, Clinical trial (phase I)

## Abstract

**Background:**

Patients with polymetastatic cancer are most often treated with systemic therapy to improve overall survival and/or delay progression, with palliative radiotherapy reserved for sites of symptomatic disease. Stereotactic ablative radiotherapy (SABR) has shown promise in the treatment of oligometastatic disease, but the utility of SABR in treating all sites of polymetastatic disease has yet to be evaluated. This study aims to evaluate the maximally tolerated dose (MTD) of SABR in patients with polymetastatic disease.

**Methods:**

Up to 48 patients with polymetastatic cancer (> 10 sites) will be enrolled on this phase I, modified 3 + 3 design trial. Eligible patients will have exhausted (or refused) standard systemic therapy options. SABR will be delivered as an escalating number of weekly fractions of 6 Gy, starting at 6 Gy × 2 weekly fractions (dose level 1). The highest dose level (dose level 4) will be 6 Gy × 5 weekly fractions. Feasibility and safety of SABR will be evaluated 6 weeks following treatment using a composite endpoint of successfully completing treatment as well as toxicity outcomes.

**Discussion:**

This study will be the first to explore delivering SABR in patients with polymetastatic disease. SABR will be planned using the guiding principles of: strict adherence to dose constraints, minimization of treatment burden, and minimization of toxicity. As this represents a novel use of radiotherapy, our phase I study will allow for careful selection of the MTD for exploration in future studies.

**Trial registration:**

This trial was prospectively registered in ClinicalTrials.gov as NCT04530513 on August 28, 2020.

**Supplementary Information:**

The online version contains supplementary material available at 10.1186/s12885-021-08020-2.

## Background

In patients who have metastatic cancer, cancer which has spread beyond the primary site to other distant sites in the body [1], the standard of care treatment is most often systemic therapy. Systemic therapy, which may include hormonal therapy, chemotherapy, targeted therapy and/or immunotherapy, has been the cornerstone of providing progression-free and overall survival advantages in patients who have metastatic cancer [2]. While systemic radiotherapy in the form of therapeutic radiopharmaceuticals are available or emerging as treatment for some polymetastatic cancers (i.e. neuroendocrine, thyroid, prostate cancer) [3], in most cases this treatment has been limited to the palliation of bone disease with bone seeking radiopharmaceuticals [4].

The traditional role of radiotherapy in patients who have metastatic cancer has been for palliation of symptomatic metastases where systemic therapy does not penetrate (i.e. brain metastases) [5], or targetable sites of disease causing symptoms such as pain, obstruction and/or bleeding [6–8]. The doses of radiation used for palliation are typically low, utilizing simple radiation planning techniques with the goal of obtaining symptom relief while minimizing treatment toxicity as well as financial toxicity and treatment burden.

Recent studies have examined a subset of patients with metastatic cancer who have a limited burden of metastatic disease, known as oligometastatic cancer. The oligometastatic state is best defined as a stage of disease where a cancer has spread beyond the primary tumour to a limited number of sites, but is not yet widely metastatic [9]. While definitions of oligometastatic cancer vary, most would consider the oligometastatic state to represent 1–3 or 1–5 metastatic lesions [10, 11].

Recent randomized trials have reported improvements in progression-free and overall survival with aggressive treatments directed at oligometastases [12–15], including stereotactic ablative radiotherapy (SABR). The application of ablative therapies for patients with 4–10 metastatic sites is being evaluated in the randomized phase III COMET-10 trial [16] where patients are randomized to ablative SABR to all sites of disease versus standard of care treatment.

In contrast to the oligometastatic state, the polymetastatic state exists beyond the oligometastatic state, where widespread dissemination of metastatic cancer has occurred (i.e. non-oligometastatic, > 10 sites of metastases) [17]. Even with recent improvements to systemic therapy options for patients who have polymetastatic cancer, it is expected that a patient’s cancer will eventually become resistant to systemic therapy, necessitating a switch to a different drug regimen. Eventually, these patients exhaust all available lines of systemic therapy and will succumb to their cancer.

As a single modality, radiotherapy is associated with higher response rates compared to most systemic therapies given with palliative intent [6]. However, it is only with recent advances in radiotherapy planning and delivery technology that SABR can be considered as potentially feasible to treat all sites of polymetastatic cancer. Given that SABR has shown promise in providing progression-free and overall survival advantages in the oligometastatic state, we postulate that the benefits of stereotactic radiotherapy may not be limited only to those with oligometastases.

The toxicity of SABR may depend primarily on the doses of radiotherapy delivered to organs at risk, rather than the number of metastatic sites treated [18]. For serial organs, such as the spinal cord, reducing the maximum point dose of radiation is expected to reduce the probability of toxicity. For parallel organs, such as the kidney and lung, reducing a critical volume of the organ receiving radiation from a specific dose of radiation is expected to reduce the probability of toxicity. For this trial, we will employ strict dose constraints for both serial and parallel structures which have been established as safe when utilizing SABR [19]. At the same time, cumulative low doses to larger volumes of tissue can be expected and may be a source of toxicity, although older literature employing hemi-body radiation suggests these doses may be tolerable [20].

Given the rationale behind expansion of the oligometastatic paradigm (i.e. with the COMET-10 trial), we believe it is reasonable to postulate that the benefits of stereotactic radiotherapy may be independent of lesion number provided the treatment can be performed without excess radiation related toxicity. In this modified phase I dose escalation trial, we will test the hypothesis that SABR can be safely delivered to all sites of polymetastatic (> 10 lesions) cancer in patients for whom standard of care systemic therapies have been exhausted (or if additional systemic therapy is refused) to determine the maximally tolerated dose (MTD).

## Methods/design

### Objective

The objective of this trial is to assess the safety and feasibility of delivering SABR to patients with polymetastatic cancer (greater than 10 sites of disease) by establishing the MTD of SABR when given at 6 Gy per fraction.

### Study design

**Fig. 1:**
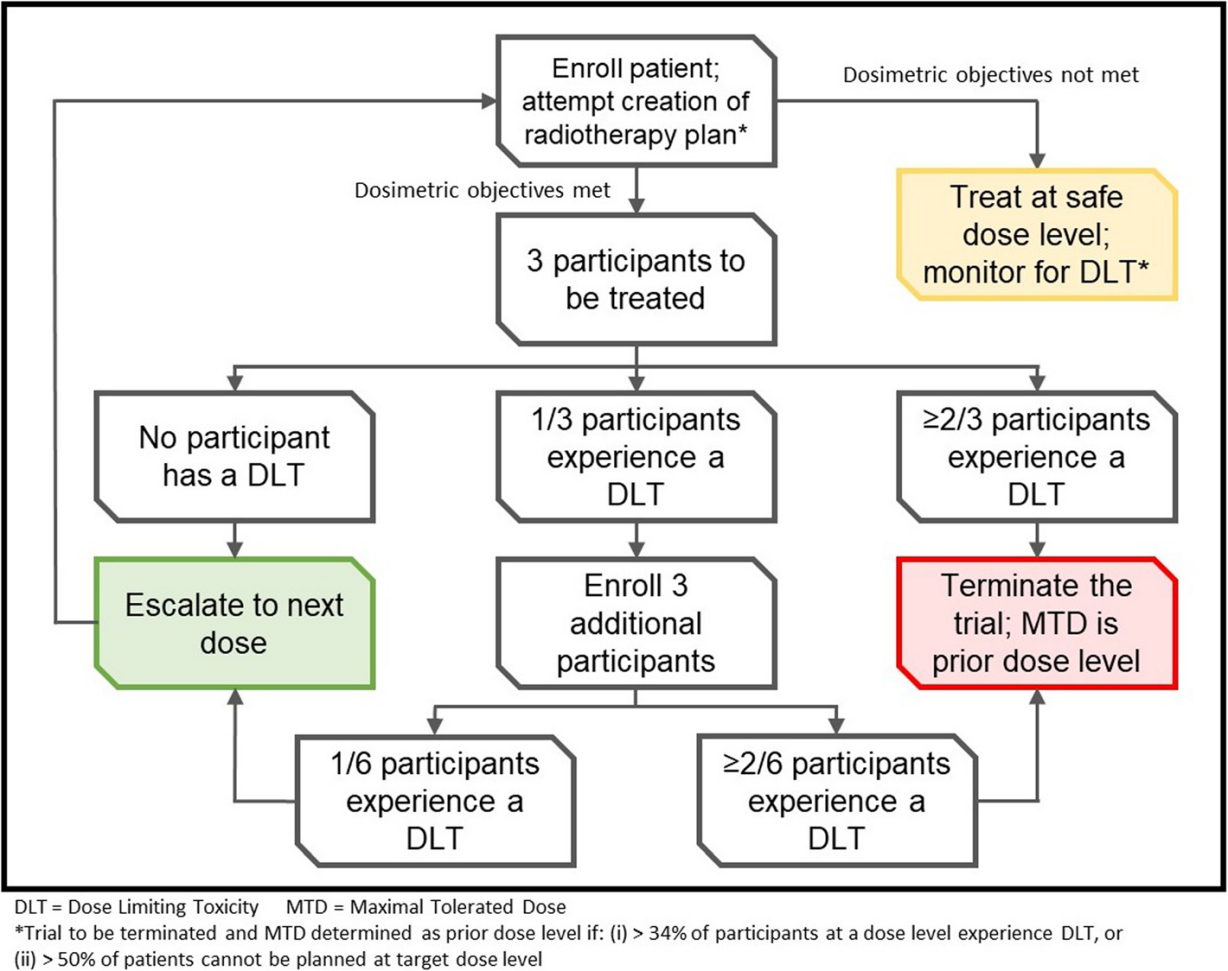
Schema of the ARREST clinical trial. The initial dose level will be 6 Gy in 2 weekly fractions, with subsequent dose escalations to be achieved by increasing the number of weekly fractions for each dose level until the maximum dose level of 6Gy in 5 weekly fractions is achieved. DLT will be assessed 6 weeks after completion of radiotherapy

This is a modified 3 + 3 design Phase I dose escalation trial. Participants will be from a single tertiary academic radiotherapy department in Canada. Three patients will initially be treated at dose level 1, 12 Gy in 2 fractions over 2 weeks, with evaluation of toxicity occurring 6 weeks after completion of treatment. If the composite co-primary endpoints are met, subsequent dose escalations will be accomplished by increasing the number of 6 Gy weekly fractions up to a maximum dose level of 30 Gy in 5 fractions over 5 weeks (Fig. 1). Given the potentially wide variety of anatomic scenarios for polymetastatic disease and the potential planning challenges beyond clinical tolerability, dosimetric planning feasibility will be evaluated with a planning success rate of greater than 33% deemed as acceptable for each dose level. Thus, a total of 12 patients per dose level could potentially be accrued to fully determine the clinical and technical feasibility.

#### Endpoints

##### Co-primary safety endpoint

For a given patient, safety of treatment at a given dose level will be determined by meeting all of the following criteria:

1. The patient successfully receives all treatment at the assigned dose level
2. No Grade 5 toxicity (patient death) attributable to treatment occurs.
3. No Grade 4 toxicity (CTCAE v4.0) at the 6-week assessment among hematologic, cardiac, hepatobiliary or respiratory domains.
4. No more than three instances of Grade 3–4 toxicities among other domains.

##### Secondary endpoints

1. The patient is successfully able to be planned at the assigned dose level.
2. Quality of life, assessed with the Functional Assessment of Cancer Therapy: General (FACT-G) and the EuroQol 5 Dimension, 5 Level (EQ-5D-5L)
3. Time to development of new metastatic lesions
4. Progression-free survival, defined as time from randomization to disease progression at any site or death
5. Overall survival, defined as time from randomization to death from any cause

#### Patient selection

##### Inclusion criteria

- Age 18 or older
- Willing and able to provide informed consent
- Eastern Cooperative Oncology Group (ECOG) performance status 0–2
- Life expectancy > 3 months
- Histologically confirmed malignancy with metastatic disease detected on imaging. A biopsy of a metastatic site is preferred, but not required
- Presence of polymetastatic disease, defined as total number of lesions greater than 10
- No standard of care systemic therapy option exists for the patient, the patient refuses further standard systemic therapy, or there is no intent to deliver systemic therapy for 3 months after enrollment
- At the discretion of the treating oncologist, it is believed that all sites of disease can be safely planned and treated

##### Exclusion criteria

- Serious medical comorbidities precluding radiotherapy. These include interstitial lung disease in patients receiving lung radiotherapy, Chron’s disease in patients where the gastrointestinal tract will receive radiotherapy, ulcerative colitis where the bowel will receive radiotherapy, or connective tissue disorders such as lupus or scleroderma.
- For patients with liver metastases, moderate/severe liver dysfunction (Child Pugh B or C)
- Inadequate baseline bone marrow function (i.e. symptomatic anemia, neutropenia and/or thrombocytopenia which may interfere with the ability to deliver radiotherapy)
- Chronic kidney dysfunction (eGFR less than 30) where a significant dose of radiation is expected to be delivered to the kidneys.
- Substantial overlap with a previously treated radiation volume. Prior radiotherapy in general is allowed, as long as the composite plan meets dose constraints herein. For patients treated with radiation previously, biologically effective dose calculations should be used to equate previous doses to the tolerances doses listed in Additional file 1.
- Prior treatment with systemic radiopharmaceuticals (i.e. Radium-223 or Lutetium-177)
- Any single lesion greater than 5 cm in size.
- More than 50 metastases (count excluding equivocal lesions < 5 mm in size)
- Any brain metastasis greater than 3 cm in size, or a total volume of brain metastases greater than 30 cc.
- Polymetastatic cancer limited to the brain.
- Brainstem metastases.
- Evidence of epidural disease on baseline imaging.
- Clinical or radiologic evidence of spinal cord compression.
- Dominant brain metastasis requiring surgical decompression.
- Inability to treat all sites of disease, which may include disease involving the gastrointestinal tract, mesenteric lymph nodes, or skin. This may also include diffuse/miliary metastatic disease (i.e. brain, bone, lung, liver) or other sites where it would be impossible to deliver radiotherapy at the intended dose level (i.e. lymphangitic spread, malignancy pleural effusion/ascites, leptomeningeal metastases).
- Systemic therapy use 2 weeks prior to radiotherapy delivery.
- Pregnant or lactating women.

#### Pre-treatment evaluation

- History and Physical examination, including prior cancer therapies.
- Restaging within 6 weeks prior to enrolment:
- Brain: CT or MRI for tumour sites with propensity for brain metastases. All patients with brain metastases (at enrolment or previously treated) require an MRI of the brain.
- Body: CT neck/chest/abdomen/pelvis and bone scan is required. This may be replaced or supplemented with an appropriate PET/CT (i.e. 18-FDG or PSMA) for tumours where uptake is expected.
- Additional imaging as clinically indicated
- Complete blood count (Hemoglobin, white blood cell count, absolute neutrophil count, platelets).
- Creatinine
- Liver function tests (AST, ALT, GGT, alkaline phosphatase), albumin, bilirubin and INR for patients with liver metastases.
- Pregnancy test for women of child-bearing age.
- Completion of FACT-G and EQ-5D-5L quality of life forms.
- Radiotherapy treatment plan has been reviewed by a physicist or radiation oncologist.

#### Data collection

All study data will be entered electronically into REDCap. Any de-identified source documents may be uploaded directly into REDCap for data entry and analysis.

#### Treatment plan

SABR in will be delivered with three major guiding principles:

1. **Minimization of Toxicity**. The radiotherapy doses used herein are lower than those used for radical treatments, and normal tissue tolerance doses will never be exceeded. Concurrent chemotherapy or targeted therapy at the time of radiotherapy (other than conventional hormone-based therapies which may continue) is not permitted. The starting dose for this trial will by 12Gy in 2 fractions with each fraction given weekly, a dose expected to be safe and well-tolerated based on prior clinical experience with hemi-body radiation.

2. **Minimization of Treatment Burden**. To avoid excessive treatment delivery time and treatment burden, radiotherapy is to be delivered using a limited number of isocentres. Given the volumes to be treated, a given “fraction” may require treatment over 1–3 days to keep daily treatment times tolerable. The smallest number of isocentres will be used and will be limited to three isocentres treated per treatment day. A maximum of three treatment days are to be utilized per dose level (i.e. three treatment days per week maximum) for a maximum of 9 isocentres per fraction. For example, a patient requiring treatment of polymetastatic disease involving the brain, lung and abdomen might require a given fraction to be delivered over 3 consecutive days: day 1, up to 3 isocentres to cover head, neck and upper thorax; day 2, up to 3 isocentres to cover lower thorax and upper abdomen; day 3, up to 3 isocentres to cover lower abdomen and pelvis.

3. **Strict adherence to dose constraints**. If a patient undergoes planning but cannot be treated at the current dose level they will receive treatment at the next highest dose level that can be safely planned. These patients will still be followed as per the clinical trial, and toxicity will be recorded. The phase 1 3 + 3 dose escalation evaluation will only apply to those who can have a safe radiotherapy treatment plan created at the target dose level. For example, a patient who fails planning at 30Gy in 5 fractions but who has a radiation plan meeting dose constraints at 18gy in 3 fractions will be treated at the lower dose level (and counted as a treatment planning failure at the higher dose level)

The fraction size throughout the study will remain at 6 Gy per fraction with dose constraints as outlined in Additional file 1. Each fraction must be completed within 3 treatment days and 1 calendar week. Dose escalation will proceed as follows, starting at Level 1; dose level 0 exists for those patients who fail planning at even dose level I and is deemed safe (based on the body of literature demonstrating safe delivery of 6Gy hemi-body radiation without conformal delivery):

##### De-escalation dose level

Dose level 0: 6Gy × 1 fraction to all sites in 1 week.

##### Escalation dose levels

Dose level 1: 6Gy × 2 fractions to all sites in 2 weeks.

Dose level 2: 6Gy × 3 fractions to all sites in 3 weeks.

Dose level 3: 6Gy × 4 fractions to all sites in 4 weeks.

Dose level 4: 6Gy × 5 fractions to all sites in 5 weeks.

If treatment at the accrued dose level cannot be planned safely, then the next lowest dose level will be attempted to be planned and delivered to the patient if it can meet organ at risk (OAR) constraints and target coverage goals. For example, if radiation cannot safely be planned at dose level 1, then dose level 0 will be attempted to be planned. For higher dose levels, multiple de-escalations can be planned until a dosimetrically safe plan is generated.

##### Target definition

The maximum number of targets will be 50, excluding indeterminate lesions. For patients with lymph node metastases, a targetable lesion will be considered as a lymph node > 10 mm in short axis or with radiographic features strongly suspicious for metastatic involvement (i.e. presence of necrosis, uptake of radiotracer in PET). Lung metastases will be considered for treatment when the short axis is > 8 mm or radiographic features are strongly suspicious for metastatic involvement (i.e. uptake of radiotracer in PET).

Targets which have previously been treated with ablative or palliative therapies (i.e. SABR, palliative radiotherapy, radiofrequency ablation, or surgery) shall not be counted unless there is unequivocal progression on baseline imaging.

Patients who have small indeterminate nodules (i.e. a 3 mm lung nodule) will not have these lesions count towards the number of target lesions and will not be targeted with SABR.

##### Simulation and immobilization

Patients will be simulated using a fast helical CT scan through the entire body, at minimum vertex of the skull to the mid femur. If there is disease below the lesser trochanter, a separate CT simulation of the lower extremities (from the top of femoral head to the bottom of the feet) will be used for treatment planning. Immobilization will be with a thermoplastic shell with arms down, a position that has been shown to be safe for lung radiotherapy [21]. Total body stereotactic immobilization is allowed.

An additional 4D-CT scan is mandatory for targets within the thorax and upper abdomen. Where indicated and tolerable, gating (preferably with a wide duty cycle) or deep inspiration breath hold will be utilized for respiratory motion management in the thorax and upper abdomen. In cases where gating or DIBH are not tolerable, a free breathing delivery will be utilized with appropriate internal target volume (ITV) margins to account for respiratory movement.

The number of isocentres should be kept to a minimum, and it is suggested to utilize a single isocentre per body region whenever possible using volumetric modulated arc therapy (VMAT) or intensity-modulated radiotherapy (IMRT) to allow simultaneous treatment of multiple lesions. However, treatment using multiple isocentres is permitted with a limit of three isocentres per treatment day (nine per fraction) if needed for optimal target coverage.

##### Volume definitions

The gross tumor volume (GTV) will be defined as the visible tumor on imaging for all lesions. No additional margin will be added for microscopic spread of disease (i.e. Clinical Target Volume (CTV) = GTV), including for vertebral body metastases. For targets expected to be impacted by respiratory motion, an ITV may be created. The Planning Target Volume (PTV) will be between 2 mm and 5 mm depending on the site of disease, immobilization and set-up accuracy.

##### Treatment planning

Treatment planning will be performed using the fast helical CT simulation scan, with all OARs contoured. If needed, the 4D dataset may be used for planning tumours in the thorax and abdomen (using the untagged or a subset average depending on the motion assessment). Dose constraints may not be exceeded under any circumstance. There is no prespecified conformity index to be used. No hot spots (> 115%) are allowed outside of the PTV, and hot spots greater than 115% are only permitted with an exemption from the study principal investigator.

The target coverage goals are listed in Table 1. Should the radiation treatment plan not meet the targets below (i.e. falls into “not acceptable” category), the patient will be considered a treatment planning failure and will not be considered for entry into the 3 + 3 dose escalation study. The patient will then be planned at the next highest dose level where an acceptable radiation treatment plan can be generated and followed per the study protocol to assess toxicity. The tumours treated on any treatment day should meet target coverage goals from the isocentres delivered on that treatment day independently of the composite total dose.

**Table 1:** Target coverage goals for the gross tumour volume (GTV) and planning target volume (PTV) on the ARREST clinical trial

**Goal**	**Acceptable**	**Not acceptable**
PTV: ≥95% of the PTV to receive ≥95% of the prescription dose for every lesion.	**GTV**: ≥95% of the GTV to receive ≥95% of the prescription dose for every lesion.	1. Failure to meet dose constraints listed in Additional file 1 2. Not being able to meet minimum “Acceptable” coverage for every lesion i.e. 3. < 90% of lesions meet the “Goal” target coverage criteria

##### Quality assurance

Each patient will have their treatment plan peer-reviewed by another radiation oncologist or at quality assurance rounds. The dose to each organ at risk must be verified by the treating physician or physicist to ensure doses to OARs are within acceptable limits. Dose delivery for VMAT or IMRT treatment plans will be confirmed prior to treatment by medical physics.

##### Special considerations during radiotherapy delivery

Given the large volume irradiated, pre-treatment with anti-emetics is mandatory for all patients on this study. The preferred treatment regimen will be ondansetron 8 mg 1–2 h prior to each radiotherapy fraction.

Patients are required to have bloodwork demonstrating adequate bone marrow function prior to delivery of the second fraction of radiotherapy, and every fraction thereafter. It is suggested this bloodwork be taken either after the last treatment of the previous level or on the first day of the next dose level, and reviewed by the treating oncologist prior to delivery of the next dose level of radiotherapy treatments. One repeat draw of bloodwork is permitted if the bloodwork values are just below the thresholds listed below. Criteria for stopping radiotherapy due to concerns of inadequate bone marrow function will be: CTCAE grade 3 anemia (hemoglobin less than 80 or symptomatic requiring a transfusion); CTCAE grade 3 thrombocytopenia (platelets less than 50), CTCAE grade 3 neutropenia (absolute neutrophil count less than 1).

Patients are not allowed to receive cytotoxic, immunotherapeutic or molecularly targeted agents within a time period commencing 2 weeks prior to initiation of radiotherapy until 6 weeks after completion of therapy. Patients on conventional hormone therapy (i.e. anti-estrogen or anti-testosterone therapy) may continue these medications.

##### Consent process

Written, informed consent will be obtained from all participants prior to enrolment. A sample informed consent from may be viewed in Additional file 2.

##### Subject discontinuation and withdrawal

Subjects may voluntarily withdraw from study participation at any time. If a subject is removed from the study, an attempt should be made to obtain laboratory and clinical evaluations that would have been obtained at the completion of the study. If the subject is removed because of an adverse event, they should remain under medical care and observation by their treating physician as long as appropriate.

##### Follow-up evaluation

Patient will be seen 6 weeks post treatment to evaluate short-term treatment toxicity. If patients cannot travel for this visit (or refuse to travel), an attempt will be made to contact the patient by phone to complete follow-up information. It must be documented if follow-up information is being gathered by telephone. The FACT-G and EQ-5D-5L quality of life questionnaire will be completed at that visit. Further follow-up will be according to physician discretion; a recommended frequency would be every 3 months at minimum. Follow-up investigations will be at the physician’s discretion. While routine reimaging is not required, a follow-up CT of the head, chest, abdomen, and pelvis at 3 months post-treatment or at the time of symptomatic progression is suggested.

#### Statistics and sample size calculation

##### Sample size

A minimum of 3 patients will be accrued. Assuming a worst case scenario where the maximum number of patients accrued cannot be planned and require additional enrolment, a maximum of 48 patients will be accrued to this Phase I dose escalation study (12 per dose level).

##### Dose escalation criteria

For a given dose level, toxicity as DLT will be assessed after three patients have been treated at a given dose level at the 6-week assessment post treatment. If all three patients meet the co-primary safety endpoint, escalation to the next dose level will occur. If two or three patients fail the co-primary safety endpoint, the MTD will be determined as the dose level preceding the failed dose level. If one patient fails the co-primary safety endpoint, an additional three patients will be accrued at that dose level. If one or more of these additional three patients fail the co-primary safety endpoint, the MTD will be determined as the dose level preceding the failed dose level. If all three additional patients meet the co-primary safety endpoint (i.e. a cumulative five of six patients pass the co-primary safety endpoint), dose escalation to the next level will occur. All patients at a given dose level must be assessed for 6-week toxicity before escalation to the next dose level can occur.

Treatment planning futility will be considered if less than 33% of the patients (to a maximum of 9 patients) at a specific dose level can successfully have a treatment plan generated for that dose level meeting organ at risk constraints. If treatment planning futility is encountered, the MTD will be considered as the dose level preceding the failed dose level.

##### Analysis plan

Descriptive statistics will be used to characterize the patient demographics on the study, distribution of lesions treated and dosimetric parameters of the treatments and toxicity experienced at 6 weeks. Progression free survival will be tracked and reported using Kaplan Meier statistics. Changes in QOL will be reported as the minimal clinically important change comparing pre and post treatment scores by patient.

##### Data and safety monitoring committee

The DSMC will meet annually after study initiation to review toxicity. The DSMC will review the chart of any case of grade 4–5 toxicity to determine if such toxicity is related to treatment. If the DSMC deems that toxicity rates are excessive (> 40% grade 4 toxicity, or any grade 5 toxicity), then the DSMC can, at its discretion, recommend cessation of the trial, dose adjustment, or exclusion of certain treatment sites and/or delivery techniques that are deemed as high-risk for complications.

##### Confidentiality of subject records

All study participant personal health information will be kept strictly confidential. All participants will be identified using initials and a unique identification number. The master list (confidential subject identification list) linking participants to their unique identification number will be kept strictly confidential by the principal investigator. Public reports of the study will not contain any names of participants.

##### Protocol amendments

The trial protocol will be amended only through approval by the principal investigator (current version: 1.0, protocol date July 28, 2020). It will be the responsibility of the principal investigator to disseminate amendments to co-investigators, the research ethics board and trial registries. Authorship of the trial abstract and manuscript will be decided by the principal investigator at the time of submission.

## Discussion

This novel phase I trial seeks to investigate the MTD of SABR delivered to patients with polymetastatic cancer who have exhausted (or refused further standard of care) systemic therapy options. We believe that initial evaluation with a rigorous phase I trial is necessary to determine what treatment dose can be safely considered in patients with polymetastatic cancer prior to embarking on evaluation of the potential efficacy of this treatment modality.

Precedent for the treatment of extensive metastatic disease with radiotherapy exists in the experience of using hemi-body radiotherapy for palliation of diffuse bone metastases [22], as well as the use of radio-ligand or systemic radiopharmaceuticals for the treatment of metastases [23]. Treating the entire body with total body radiation is not feasible due severe bone marrow and gastrointestinal tract dysfunction, side effects seen when total body irradiation is purposefully given as part of a stem cell transplant [24]. However, with modern radiation planning software and delivery platforms, we believe it is feasible and practical to give SABR to all sites of polymetastatic disease while minimizing doses to organs at risk.

Further rationale for exploring SABR in the polymetastatic setting comes from emerging randomized evidence that supports aggressive radiotherapy to the primary tumour, either with SABR or conventional (chemo) radiation in patients newly diagnosed with polymetastatic prostate [25] and nasopharyngeal cancers [26]. For these tumour types, improvements in progression-free and overall survival have been observed with reasonable side effect profiles. Sites of oligometastatic/polymetastatic disease were not routinely treated with radiotherapy in these studies, leading to the hypothesis that outcomes could possibly be improved further with the addition of SABR, assuming it can be safely delivered.

In designing this trial, we chose to evaluate dose levels in incremental fractions of 6 Gy for several reasons. First, this dose level represents familiarity in the context of hemi-body radiotherapy. While hemi-body radiotherapy has fallen out of favour due to enhancements in systemic therapy and improvements in radiopharmaceuticals, the experience of using this technique serves as the rationale behind using 6 Gy fractions of SABR. The starting dose of 12 Gy in 2 weekly fractions should also be expected to offer palliation of symptomatic metastases. Second, by escalating the number of fractions but keeping the dose per fraction consistent, an optimal radiotherapy treatment plan can be generated for the target dose level. If this optimal treatment plan does not to meet dose constraints at the target dose level, the number of fractions can be easily decreased without needing to generate a new radiotherapy plan. This allows for increased efficiency of workflow in a setting where we anticipate treatment plan generation will be resource intensive and time consuming. Third, delivering the fractions once per week allows for careful evaluation of potential, unanticipated toxicity during treatment. For example, we are carefully evaluating for the possibility of bone marrow suppression due to SABR, particularly in our chosen patient population who have exhausted (or refuse) further lines of systemic therapy and may have pre-existing myelosuppression. Finally, while the doses of SABR within this trial would not typically be considered below truly ablative doses, we believe the maximum dose level will strike an acceptable balance between anticipated efficacy, treatment intent, and potential toxicity.

Our study population was carefully considered for creation of this novel phase I trial. We chose to evaluate a subset of patients where systemic therapy use at the time of SABR is unlikely to interfere acutely with the evaluation of the MTD, as no standard systemic therapy options are available (or are refused by the patient). It is also unlikely that any toxicity from SABR in this study would interfere with the possibility of receiving subsequent systemic therapy that may become available or the patient chooses to access. However, patients enrolled on this study are expected to have been heavily pretreated with systemic therapy, which may influence secondary endpoints in our trial such as progression-free survival and overall survival. Some have argued phase I trials should be offered earlier in the treatment pathway for patients [27], though we believe the toxicity of SABR in this setting needs to be carefully evaluated before earlier implementation can be considered. Whether there is an upper limit to the number of lesions that can safely be targeted in SABR remains to be seen. For this trial, we have arbitrarily chosen 50 targets. More likely, it is the relative distribution, size, and proximity of metastases to critical organs at risk that will determine the maximum number of lesions that can safely be treated. Valuable information regarding the variety of anatomic scenarios when considering polymetastatic disease will be gained through this experience.

## Conclusion

ARREST is a novel phase I trial leveraging the availability of modern image guided radiotherapy for the treatment of polymetastatic disease. Such treatment, if shown to be safe and tolerable, may provide additional treatment options for patients with extensive metastases who are ineligible or unwilling to undertake palliative systemic therapies.

## Supplementary Information

**Additional file 1.** Dose constraints for treatment planning.

**Additional file 2.** Sample consent form.

## Data Availability

Not applicable.

## Abbreviations

18-FDG: 18-flurodeoxyglucose
4D-CT: Four-dimensional computed tomography
ALT: Alanine aminotransferase
AST: Aspartate aminotransferase
CT: Computed tomography
CTCAE: Common toxicity criteria for adverse events
CTV: Clinical target volume
eGFR: Estimated glomerular filtration rate
EQ-5D-5L: EuroQol 5 Dimension, 5 Level
FACT-G: Functional assessment of cancer therapy: general
GGT: Gamma glutamyl transpeptidase
GTV: Gross tumour volume
IMRT: Intensity modulated radiotherapy
INR: International normalized ratio
ITV: Internal target volume
MRI: Magnetic resonance imaging
MTD: Maximally tolerated dose
PSMA: Prostate specific membrane antigen
PTV: Planning target volume
SABR: Stereotactic ablative radiotherapy
VMAT: Volumetric modulated arc therapy
