# Supplementary material for: Ablative radiation therapy to restrain everything safely treatable (ARREST): study protocol for a phase I trial treating polymetastatic cancer with stereotactic radiotherapy
Source: BMC Cancer. 2021 Apr 14;21:405. doi: 10.1186/s12885-021-08020-2 (PMC8048078; doi:10.1186/s12885-021-08020-2)
Supplement: Supplementary file 1 — Additional file 1. Dose constraints for treatment planning. [file 12885_2021_8020_MOESM1_ESM.docx]

# ADDITIONAL FILE 1: DOSE CONSTRAINTS

These are based on the NRG-LU002 trial and the SABR-COMET trial. If any structure is not listed, the constraints may be calculated using the linear quadratic formula from accepted QUANTEC doses, using an alpha-beta ratio of 2 for late effects.

**Table A1: Dose Constraints for Serial Structures.** D0.03cc = maximum dose in Gy allowable to the hottest 0.03 cc; other D values are used in the same way

| **Structure** | **Volume** | **1 Fraction** | **2 Fractions** | **3 Fractions** | **4 Fractions** | **5 Fractions** |
| --- | --- | --- | --- | --- | --- | --- |
| **Optic Pathway** | D0.03cc | 10 | 14.4 | 19.5 | 22.4 | 25 |
| **Cochlea** | D0.03cc | 9 | 14 | 18 | 22.4 | 25 |
| **Brainstem** | D0.03cc | 15 | 19 | 23.1 | 28 | 31 |
|  | D0.5cc | 10 | 13.2 | 18 | 22.4 | 25 |
| **Brain minus PTV** | D50% | 6 | 9 | 12 | 13.5 | 15 |
| **Spinal Cord (applied to Canal or Cord PRV, see notes in 6.2.6)** | D0.1cc (for entire structure) | 14 | 17 | 22.5 | 24.8 | 28 |
|  | D0.03cc (to each segment +/- 1 vertebral body) | 14 | 17 | 22.5 | 24.8 | 28 |
|  | D0.35cc (to each segment +/- 1 vertebral body) | 10 | 13.2 | 18 | 22.4 | 25 |
| **Cauda Equina or Sacral Plexus** | D0.03cc | 16 | 18.5 | 22.5 | 28.4 | 31.5 |
|  | D5cc | 14 | 18 | 21.9 | 27.2 | 30 |
| **Esophagus** | D0.03cc | 15.4 | 20 | 25.2 | 30.7 | 35 |
|  | D5cc | 11.9 | 15 | 17.7 | 23.8* | 27.5 |
| **Brachial Plexus** | D0.03cc | 16.4 | 20 | 26 | 27.2 | 32.5 |
|  | D3cc | 13.6 | 18 | 22 | 25.5 | 27 |
| **Heart** | D0.03cc | 20* | 21 | 30 | 34 | 38 |
|  | D15cc | 16 | 20 | 24 | 29 | 32 |
| **Great Vessels** | D0.03cc | 37 | 37 | 45 | 46 | 53 |
|  | D10cc | 31 | 32 | 39 | 42 | 47 |
| **Proximal Bronchial Tree** | D0.03cc | 20.2 | 25 | 30 | 36 | 40 |
|  | D4cc | 17.4 |  | --- | --- | --- |
|  | D5cc | --- | 23 | 25.8 | 29 | 32 |
| **Chest Wall or Rib** | D0.03cc | 33 | 40 | 50 | 50 | 57 |
|  | D5cc | 28 | 32 | 40 | 40 | 45 |
| **Skin** | D0.03cc | 27.5 | 27.5 | 33 | 34 | 38.5 |
|  | D10cc | 24* | 25 | 31 | 32 | 36.5 |
| **Stomach** | D0.03cc | 16 | 20 | 24 | 29 | 32 |
|  | D10cc | 13 | 17.5 | 21 | 24 | 26.5 |
| **Bile Duct** | D0.03cc | 30 | 30 | 36 | 36 | 41 |
| **Duodenum** | D0.03cc | 17 | 20 | 22.2 | 23.6 | 26 |
|  | D10cc | 9 | 13 | 18 | 19 | 21 |
| **Jejunum or Ileum (Bowel Bag)** | D0.03cc | 22 | 20 | 27 | 29 | 32 |
|  | D30cc | 12.5 | 14.5 | 18 | 19 | 21 |
| **Colon or Rectum** | D0.03cc | 27* | 28 | 34.5 | 36 | 40 |
|  | D20cc | 18 | 20 | 24 | 26 | 28.5 |
| **Ureter** | D0.03cc | 30* | 32 | 40 | 40 | 45 |
| **Bladder** | D0.03cc | 20 | 27 | 33 | 34 | 38 |
|  | D15cc | 12 | 17 | 21 | 24 | 27.5 |
| **Penile Bulb** | D3cc | 16 | 20.5 | 25 | 27.2 | 30 |
| **Femoral Heads (if uninvolved)** | D10cc | 15 | 20 | 24 | 27.2 | 30 |

**Table A2: Dose Constraints for Parallel Structures.** Parallel structures require the use of a ‘critical volume’ (CV), also termed a ‘complementary volume’. For example, for lung, the CV1500cc is listed as 12.5 Gy for 5-fraction treatments, meaning that there must be 1500 cc of lung receiving 12.5 Gy or less. This is read from the left-hand side of a DVH. For further information on calculating the CV, with DVH examples, see *Application of Critical Volume-Dose Constraints for Stereotactic Body Radiation Therapy in NRG Radiation Therapy Trials* at <https://www.redjournal.org/article/S0360-3016(17)30241-9/abstract>. The VX refers to the percent of lung (minus GTVs) receiving X Gy or more.

| **Structure** | **Volume** | **1 Fraction** | **2 Fractions** | **3 Fractions** | **4 Fractions** | **5 Fractions** |
| --- | --- | --- | --- | --- | --- | --- |
| **Lung (combined right and left, subtract GTVs if applicable)** | CV1500cc (Gy) | 7 | 8.75 | 10.5 | 11.5 | 12.5 |
|  | V8Gy(%) | 37 |  |  |  |  |
|  | V9.5Gy (%) |  | 37 |  |  |  |
|  | V11Gy(%) |  |  | 37 |  |  |
|  | V12.25Gy (%) |  |  |  | 37 |  |
|  | V13.5Gy(%) |  |  |  |  | 37 |
|  | V10Gy(%) | 15 |  |  |  |  |
|  | V14Gy (%) |  | 15 |  |  |  |
|  | V16.5Gy(%) |  |  | 15 |  |  |
|  | V18Gy (%) |  |  |  | 15 |  |
|  | V20(%) |  |  |  |  | 15 |
|  | V3.25Gy(%) | 80 |  |  |  |  |
|  | V4Gy (%) |  | 80 |  |  |  |
|  | V4.5Gy(%) |  |  | 80 |  |  |
|  | V4.75Gy (%) |  |  |  | 80 |  |
|  | V5(%) |  |  |  |  | 80 |
| **Liver (subtract GTVs if applicable)** | CV700cc (Gy) | 11 | 14 | 17.1 | 19 | 21 |
| **Kidney cortex (combined left and right, subtract GTVs if applicable)** | CV200cc (Gy) | 9.5 | 12.25 | 15 | 16.5 | 18 |
