# Supplementary material for: Ablative radiation therapy to restrain everything safely treatable (ARREST): study protocol for a phase I trial treating polymetastatic cancer with stereotactic radiotherapy
Source: BMC Cancer. 2021 Apr 14;21:405. doi: 10.1186/s12885-021-08020-2 (PMC8048078; doi:10.1186/s12885-021-08020-2)
Supplement: Supplementary file 2 — Additional file 2. Sample consent form. [file 12885_2021_8020_MOESM2_ESM.docx]

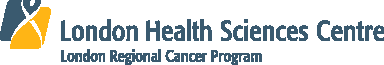


800 Commissioners Road East

PO Box 5010, STN B

London, Ontario N6A 5W9

Tel 519.685-8500

**Letter of information and consent**

**ARREST – A Phase 1 study of SABR for poly-metastatic disease**

| **Principal Investigator/**  **Investigator Conducting Study:** | Dr. Glenn Bauman, MD, FRCPC  (519) 685-8600 ext. 53177 |
| --- | --- |
|  |  |
| **Funding Source:** | This trial is funded by a grant from the Ontario Institute for Cancer Research and local research funds from the Department of Oncology |

**Emergency Contact Number** (24 hours / 7 days a week): If a medical emergency arises, proceed to your local Emergency Department. The emergency physician can contact the oncologist on call at 519-685-8500 if required.

Non-Emergency contact numbers are at the end of this document under Contacts.

In this Letter of Information document, “you” always refers to the study participant. If you are a substitute decision maker (SDM) (i.e. someone who makes the decision of participation on behalf of the participant), please remember that “you” refers to the study participant. If a SDM is needed for this study, you will be asked to review and sign the consent form on behalf of the participant.

**Introduction**

You are being invited to participate in a clinical trial (a type of study that involves research). Clinical trials only include participants who choose to take part. You are invited to participate in this trial because you have cancer that has spread to other parts of your body (metastatic cancer). When cancer has spread to more than 10 locations, this is called poly-metastatic disease.

This letter of information and consent form provides you with information to help you make an informed choice. Please read this document carefully and take your time in making your decision. You may find it helpful to discuss it with your friends and family.

Taking part in this study is voluntary. You may choose not to take part or if you choose to participate may leave the study at any time without giving a reason. Deciding not to take part or deciding to leave the study later will not result in any penalty or any loss of benefits to which you are entitled.

**Background**

In all cases, poly-metastatic cancer is not considered a curable form of cancer and treatments are given with the goal of trying to control the cancer for a time and reduce symptoms from the cancer. The usual treatment for poly-metastatic cancer is drug therapy (such as chemotherapy or hormone therapy) to try to control the cancer and reduce symptoms. Radiation treatment is sometimes used to treat isolated spots of cancer that are causing symptoms but has not traditionally been directed to all sites of cancer because of concerns about the side effects of treating many sites at once in the body.

Unfortunately, for many people, over time, drug treatments may lose their effectiveness in treating the cancer or people may develop side effects or may not be able to tolerate ongoing drug treatments because of effects of the drugs on the body. For people in this situation, treatment options are limited and often focus on managing the symptoms of cancer such as treating pain with pain medications without active treatment of the cancer.

Stereotactic ablative radiotherapy (SABR) is a new radiation treatment that delivers high-dose, precise radiation to small areas in the body. This new technique can potentially allow radiation treatments to be focused more precisely, and be delivered more accurately than with older treatments. This improvement could help by reducing side effects overall (through radiation exposure to a smaller area of the body over a shorter time period), and by improving the chance of controlling the cancer by more precisely treating the cancer and by giving higher doses of radiation. SABR is considered a standard treatment for some lung cancers, and selected cancers that have spread to the brain. Ongoing studies are evaluating the use of SABR for treating people with up to 10 sites of cancer in the body, but its safety and value for treating patients with poly-metastatic cancer (more than 10 sites of cancer) is not yet known.

**Purpose**

The purpose of this study is to determine the safety of using SABR to treat people with poly-metastatic disease. To our knowledge, this is the first time that SABR will be tested in people who have poly-metastatic disease.

**Expected Number of Participants**

Up to 48 people from the London Regional Cancer Program will take part in this study. The study will take about 3 years to complete and the results should be known in about 5 years.

Your study doctor will be informed of the results of this study once they are known.

**Assignment to a Group**

If you decide to participate, you will be assigned to receive a dose of SBRT in one of the dose groups listed below. The group you are assigned to is determined based on the number of participants who have already completed study treatment and the side effects that have been observed. You will be told which treatment you are to get.

The dose groups are ordered from A-E, with A representing the least-potent dose and the Group E representing the most potent dose. In this study, participants will first be enrolled into Group B. After the first few patients are enrolled, depending on their side effects, other participants may be enrolled into a group with a higher dose (Groups C-E) or lower dose (Group A).

|  | **Overall dose (Gray)*** | **Treatment**  **frequency** | **Total length of treatment** |
| --- | --- | --- | --- |
| **Group A** | 6 | 1-3 days each week | 1 week |
| **Group B** | 12 | 1-3 days each week | 2 weeks |
| **Group C** | 18 | 1-3 days each week | 3 weeks |
| **Group D** | 24 | 1-3 days each week | 4 weeks |
| **Group E** | 30 | 1-3 days each week | 5 weeks |

*Gray/Gy is a unit of radiation

Each day, the radiation treatment takes about 20 minutes, or sometimes longer if we have to track your breathing during treatment. You will need to have a specialized computerized tomography (CT) scan to plan the radiation treatment and track your breathing movement before starting radiation treatment. This is done as part of standard of care.

**Non-Experimental Procedures**

The following tests will be done as part of this study. These tests are done as part of your standard care, in which case the results may be used. Some of these tests may be done more frequently than if you were not taking part in this study and some may be done solely for the purpose of the study. If the results show that you are not able to continue participating, your study doctor will let you know:

- history and physical examination
- routine blood tests – complete blood count (CBC) and liver function tests
- pregnancy test for women of child-bearing potential
- Full Body computed tomography (CT) scan or magnetic resonance imaging (MRI) A CT scan is a series of x-rays of the body from many angles that are turned into 3-dimensional pictures on a screen. CT scans often involve injecting a dye into your vein. An MRI is an imaging technique that uses a strong magnet to produce pictures of areas inside the body. MRI is useful for assessing organs and other soft tissue, such as the inside of bones. Depending on what kind of cancer you have, you may also need to have a CT or MRI of the brain.
- Full body PET scan: A PET scan is used to try to find small deposits of cancer that can’t be detected on CT scans, using a special radioactive material that is called a “tracer”. It involves injecting a small amount radioactive tracer into your vein, and this tracer is taken in by cancer cells. The location of the tracer in the body tells your doctor where cancer cells might be. This may not be required for all participants.
- bone scan – a type of imaging procedure that involves injecting dye into your vein and then taking a series of pictures of all of your bone.

**Experimental Procedures**

The following procedures are considered experimental and will only be done for participants on this study:

- SABR treatment

**Questionnaires:**

You will be provided with two questionnaires before starting this study, and then again 5-6 weeks after completion of your SABR treatment. The purpose of the questionnaires is to understand how your treatment and illness affects your quality of life. These questionnaires ask about how you are feeling and take about 10 minutes to complete.

These questionnaires may be completed via paper at your clinic visit, by telephone with the study coordinator, complete and return to the clinic by mail or as an online survey using REDCap if you choose to provide your email address. REDCap is an online data collection tool that is being used to collect data (responses to questions) for the study. You can be sent the questionnaires as surveys if you would like to complete the questionnaires online from home, or, you can complete the questionnaires by paper and the research coordinator will enter your data into the database.

No personal identifiers, aside from your participant study id, will be submitted with your responses. Your email address will not be used for any other purpose and will remain under secure access at the study site.

The information you provide is for research purposes only and will remain strictly confidential. Some of the questions are personal; you can decide not to answer these if you wish.

**Length of Participation**

You will be asked to come back to the clinic 5-6 weeks after you complete SABR treatment. After that, you will continue follow up visits at a schedule determined by your doctor. Study staff will collect information on your disease status and further cancer treatments you have received every 3 months up to 2 years. This information may be obtained from hospital/clinic visits or by telephone contact.

**Summary of Treatments, Tests and Procedures**

| **Assessments** | **Baseline (before you receive radiation)** | **Every week during radiation treatment** | **5-6 weeks after radiation completion** | **Every 3 months until 2 years** |
| --- | --- | --- | --- | --- |
| **Medical History** | X |  |  |  |
| **Physical Exam** | X | X | X |  |
| **Imaging of neck, chest, abdomen and pelvis and sometimes brain** | X |  |  |  |
| **Blood Tests** | X | X |  |  |
| **Symptom Assessment** | X | X | X |  |
| **Questionnaires** | X |  | X |  |
| **Follow up data collection** |  |  |  | X |

**Responsibilities**

If you choose to participate in this study, you will be expected to:

- Tell your study doctor about your current medical conditions;
- Tell your study doctor about all prescription and non-prescription medications and supplements, including vitamins and herbals, and check with your study doctor before starting, stopping or changing any of these. This is for your safety as these may interact with the treatment you receive on this study;
- Tell your study doctor if you are thinking about participating on another research study;
- Return any questionnaires that were completed to the clinic/hospital;
- Tell your study doctor if you become pregnant or father a child while participating on this study

**Risks of Participation**

Participating in this study will put you at risk for the side effects listed below. You should discuss these with your study doctor. As with any treatment additional unexpected and sometimes serious side effects are a possibility.

Your study doctor will monitor you closely to see if you have side effects. When possible other drugs will be given to you to make side effects less serious and more tolerable. Many side effects go away shortly after treatment is stopped but in some cases side effects can be serious, long-lasting, permanent, or even cause death.

If you experience serious side effects that require treatment between regular clinic/hospital visits, it is important that you make every effort to return to the clinic/hospital where your study treatment was given. If you need immediate treatment and are unable to return to the clinic/hospital where you received your study treatment, you should go to the nearest medical clinic/hospital and tell them that your study doctor should be contacted as soon as possible.

You will undergo a CT scan, called a “simulation”, to design the radiation. This CT scan is considered standard for radiation treatment and exposes you to a small amount of radiation.

Risks and side effects related to radiation are listed below.

**Risks and side effects related to radiation therapy depend on the area being treated**. For example, for a patient receiving radiation to the brain, the side effects related to the lungs and bowels do not apply.

**Very likely [Common] (*21% or more, or higher than a 1 in 5 risk)*:**

- Fatigue
- Skin Rash in area being treated
- Hair loss in area being treated

**Less likely [*Occasional*] (*5 to 20% or between a 1 in 5 and 1 in 20 risk*):**

- Nausea/vomiting
- Decreased hearing or irritation of the ears
- Dryness or irritation of the eyes
- Dry or sore mouth or throat or loss of taste during radiation treatments.
- Temporary lung injury resulting in shortness of breath or cough (if the lungs are being treated)
- Temporary difficulty or painful swallowing
- Diarrhea or cramping of the bowels (if the abdomen or pelvis are treated)
- Discomfort or frequency of urination (if the pelvis is treated)

**Rarely (*1 to 4% or between a 1 in 25 and 1 in 100 risk*):**

- Permanent lung injury resulting in shortness of breath or cough (if the lungs are being treated)
- Bone injury resulting in a broken bone (if a bone is treated)
- Changes in thinking or memory (if the brain is treated).
- Persistent cramping, diarrhea or bleeding from the bowel (if the abdomen or pelvis is treated)
- Persistent frequency or discomfort with urination
- Persistent pain in a bone, muscle, or nerve

**Rare but Serious (less than 1% or less than a 1 in 100 risk)**

Radiation treatments are associated with a small risk of serious injury to tissues or organs that are included in the area being treated. This injury may show up months to years post treatment. In very rare instances, these side effects may result in death. Some of these side effects include (depending on whether these areas are being treated):

- - Brain injury resulting in loss of strength, sensation or thinking ability
  - Spinal cord injury resulting in paralysis of the lower half of the body including both legs
  - Esophagus injury resulting in difficulty swallowing
  - Heart injury resulting in a heart attack or fluid collection around the heart
  - Rectal or bowel injury resulting in bleeding or perforation (hole in the lining of the bowel and/or rectum) or fistula (abnormal connection between the bowel and another organ)
  - Bladder injury resulting in bleeding or perforation (hole in the lining of the bladder or fistula (abnormal connection between the bladder and another organ)
  - Development of a second cancer in the radiation area, usually several years after treatment.

Your study doctor will monitor your therapy and make adjustments to your treatment or prescribe medicines in order to manage side effects that occur during treatment. The radiation technique, daily dose and total dose of radiation for your treatment will be prescribed by your study doctor in order to minimize the chance of late serious injury as outlined above.

It is possible, although unlikely, that SABR may be associated with unexpected side effects that are not yet known or included on this list. For example, when SABR was first implemented, treatment of tumors in the center of the chest was associated with a high risk of injury to the breathing passages (bronchi), and so the doses delivered to tumors in that area have been lowered to reduce that risk.

The risks and side-effects of the standard or usual treatment will be explained to you as part of your standard care and are therefore not listed.

It is possible that other drugs (prescription and non-prescription drugs), vitamins, or herbals can interact with the radiation used in this study. This can result in either the radiation not working as expected or result in severe side effects.

**Reproductive Risks**

Radiation therapy may be harmful to an unborn baby (fetus). You must not become pregnant or father a baby while receiving radiation therapy and for 6 months after the last dose. Your study doctor will discuss methods with you to ensure you do not become pregnant or father a baby during the study.

Women should not nurse (breastfeed) a baby while receiving radiation therapy and for 6 months after the last dose because the radiation used in this study might be present in breast milk and could be harmful to a baby.

If you become pregnant or father a child during this study or for 6 months after you stop receiving radiation therapy, then you should immediately notify your study doctor and you will no longer be able to participate in the study.

**Benefits**

If you agree to take part in this study, the experimental treatment may or may not be of direct benefit to you. Your cancer may shrink but these things cannot be predicted for you. The researchers hope the information learned from this study will help other patients in the future.

**Early End to Participation**

The researchers can take you off the study treatment early for reasons such as:

- The treatment does not work for you and your cancer comes back or gets worse.
- You are unable to tolerate the study treatment
- You no longer wish to participate.
- You are unable to complete all required study procedures
- New information shows that the study treatment is no longer in your best interest.
- Your study doctor no longer feels this is the best treatment for you.
- The study doctor decides to stop the study
- The Research Ethics Board withdraws permission for the study to continue
- You become pregnant

**Voluntary Participation**

Your participation in this study is voluntary. You may decide not to be in this study, or to be in the study now and then change your mind later. You may leave the study at any time without affecting your care. We will give you new information that is learned during the study that might affect your decision to stay in the study.

**Alternative Treatments**

You do not have to take part in this study in order to receive treatment/care. Other options (in addition to the standard or usual treatment described above) may include, but are not limited to:

- No therapy at this time.
- Standard of care treatment that could include: conventional radiotherapy, chemotherapy agents or other drug treatments like immunotherapy or targeted treatments, if they are appropriate for your type of cancer.
- Palliative care or Best Supportive Care (BSC). This type of care helps reduce pain, tiredness, appetite problems and other problems caused by the cancer. It does not treat the cancer directly, but instead tries to improve how you feel. Best Supportive Care tries to keep you as active and comfortable as possible.
- Other experimental studies may be available if you do not take part in this study.

Please talk to your study doctor or usual cancer doctor about the known benefits and risks of these other options before you decide to take part in this study. Your usual cancer doctor can also discuss with you what will happen if you decide not to undertake any treatment at this time.

**Rights**

You will be told, in a timely manner, about new information that may be relevant to your willingness to stay in this study.

If you decide to stop participating in the study or if your participation has been stopped, your study doctor will discuss other options with you and continue to treat you with the best means available.

You may withdraw your permission to use your personal health information for this study at any time by letting the study doctor know. However, this would also mean that you withdraw from the study. Your study data that was recorded before you withdrew will be used but no information will be collected or sent to the Clinical Cancer Research Program, LHSC, who is coordinating this study, after you withdraw your permission.

Your rights to privacy are legally protected by federal and provincial laws that require safeguards to ensure that your privacy is respected.

By signing this form, you do not give up any of your legal rights against the investigators, sponsor or involved institutions for compensation, nor does this form relieve the investigators, sponsor or involved institutions of their legal and professional responsibilities.

You will be given a copy of this signed and dated consent form prior to participating in this study.

**Costs**

The study treatment will be provided to you free of charge while you are participating in this study. The costs of your medical treatment will be paid for by your provincial medical plan to the extent that such coverage is available. There may be extra costs that are not covered by your medical plan that you will have to pay yourself; some examples may be physiotherapy or certain pain medications.

Taking part in this study may result in added costs to you (i.e. transportation, parking meals, or unpaid leave from work). You may have to pay for medication prescribed to treat or prevent side effects, and you may have to visit the hospital more often than if you were not participating in this study.

**Compensation**

You will not be paid for taking part in this study.

Should you be required to visit the centre more frequently than if you were receiving standard treatment (not participating in this study), you will be reimbursed for your parking expenses for these extra visits.

In the case of research-related side effects or injury, medical care will be provided by your doctor or you will be referred for appropriate medical care.

**Confidentiality**

Qualified and authorized representatives of the following organizations may look at your original (identifiable) medical/clinical study records at the site where these records are held for quality assurance (to check that the information collected for the study is correct and follows proper laws and guidelines). Examples include:

- Staff of the Clinical Cancer Research Unit at the London Regional Cancer Program, the research group coordinating this study under the direction of Dr. Glenn Bauman, at the LRCP
- The Quality Assurance and Education Officers from Lawson Health Research Institute (Lawson) may audit this research study for quality assurance purposes
- Western University Health Sciences Research Ethics Board that oversees the ethical conduct of this study

Records identifying you at this centre will be kept confidential and, to the extent permitted by the applicable laws, will not be disclosed or made publicly available, except as described by this consent document.

Authorized representatives of the Clinical Cancer Research Unit, London Health Sciences Centre will **receive** information related to the study, electronically, from your medical/clinical study records for quality assurance and data analysis. Your name or other information that may identify you will not be used. The records received by these organizations may contain your Study ID number, first and last initials, month and year of your birth date and sex.

All data that will be collected from this study will be considered confidential. We will maintain your confidentiality by using a unique identifier number on all documents instead of your name. A separate secure document will contain the linkage between your name and identifier number in order to minimize the possibility of a breach of your privacy. This list will be kept in a secure place, separate from your study file in the Clinical Research Unit at the London Regional Cancer Program, Lawson Health Research Institute. Your research records will be stored in a locked cabinet at the clinical trials unit. Once the data has been put into the research database, any identifying information, apart from your initials and a unique study number, will be removed from the database in order to protect your confidentiality.

All of the organizations listed in the above confidentiality sections are required to have strict policies and procedures to keep the information they see or receive about you confidential, except where disclosure may be required by law. The study doctor will ensure that any personal health information collected for this study is kept in a secure and confidential location at the London Regional Cancer Program, London Health Sciences Centre for 15 years as required by law. There are federal and provincial laws that these organizations must comply with to protect your privacy.

If the results of this study are published, your identity will remain confidential. It is expected that the information collected during this study will be used in analyses and will be published and/or presented to the scientific community at meetings and in journals.

Even though the likelihood that someone may identify you from the study data is very small, it can never be completely eliminated.

A copy of this signed and dated consent form may be included in your health record/hospital chart.

Your family doctor/health care provider will be informed that you are taking part in a study so that you can be provided with appropriate medical care. If you do not want your family doctor/health care provider to be informed, please discuss with your study doctor. For safety reasons, should you not want your family doctor/health care provider to be informed, you will not be able to participate in this study.

**Registration of Clinical Trials**

A description of this clinical trial will be available on http://www.clinicaltrials.gov. This website will not include information that can identify you. At most, the Web site will include a summary of the results. You can search this website at any time.

**Conflict of Interest**

There are no conflicts of interest to declare related to this study. The doctor treating you may also be the doctor in charge of the study.

**Contacts**

If you have questions about taking part in this study, or if you suffer a research-related injury, you should talk to your study doctor. Or, you can meet with the doctor who is in charge of the study at this institution. That person is:

Dr. Glenn Bauman 519-685-8600 ext. 53177

|  |  |  |  |
| --- | --- | --- | --- |

If you have questions about your rights as a research participant or the conduct of the study, you may contact the Patient Relations Office at LHSC at (519) 685-8500 ext. 52036.


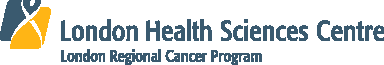


800 Commissioners Road East

PO Box 5010, STN B

London, Ontario N6A 5W9

Tel 519.685-8500

**Consent Form**

*ARREST – A Phase 1 study of SABR for poly-metastatic disease*

I have read the Letter of Information, have had the nature of the study explained to me and I agree to participate. All questions have been answered to my satisfaction. I consent to the study team to use and share my health information as described in this form. I will receive a copy of the signed letter of information and this consent form.

**To be signed and dated by the study participant**

___________________________________ ____________________________
Signature of study participant Date of Signature

__________________________________
Printed name of study participant (BLOCK CAPITALS)

___________________________________ ____________________________
Signature of person conducting the Date of Signature

informed consent discussion

__________________________________
Printed name of person conducting the

informed consent discussion (BLOCK CAPITALS)

Your signature on this form indicates that you are acting as a substitute decision maker(s) for the participant and the study has been explained to you and all your questions have been answered to your satisfaction. You agree to allow the person you represent to take part in the study. You know that the person you represent can leave the study any time.

________________________ ____________________ ________________________

Print Name of Substitute Signature Date (DD-MMM-YYYY)

Decision Maker

________________________

Relationship to Participant

Was the participant assisted during the consent process?  YES  NO

If YES, please check the relevant box and complete the signature space below:

The person signing below acted as a translator for the participant during the consent process and attests that the study as set out in this form was accurately translated and has had any questions answered.

_______________________ _______________ ___________________

Print Name of Translator Signature Date (DD-MMM-YYYY)

________________

Language
